# Crosstalk between Tryptophan Metabolism via Kynurenine Pathway and Carbohydrate Metabolism in the Context of Cardio-Metabolic Risk—Review

**DOI:** 10.3390/jcm10112484

**Published:** 2021-06-04

**Authors:** Małgorzata Kiluk, Janina Lewkowicz, Dariusz Pawlak, Anna Tankiewicz-Kwedlo

**Affiliations:** 1 Department of Internal Medicine and Metabolic Diseases, Medical University of Bialystok, 15-089 Białystok, Poland; malgorzatakiluk@gmail.com (M.K.); janina.lewkowicz@umb.edu.pl (J.L.); 2 Department of Pharmacodynamics, Medical University of Bialystok, 15-089 Białystok, Poland; dariusz.pawlak@umb.edu.pl; 3 Department of Monitored Pharmacotherapy, Medical University of Bialystok, 15-089 Białystok, Poland

**Keywords:** kynurenine pathway, diabetes mellitus type 2, diabetes mellitus type 1, cardiovascular system, review

## Abstract

Scientific interest in tryptophan metabolism via the kynurenine pathway (KP) has increased in the last decades. Describing its metabolites helped to increase their roles in many diseases and disturbances, many of a pro-inflammatory nature. It has become increasingly evident that KP can be considered an important part of emerging mediators of diabetes mellitus and metabolic syndrome (MS), mostly stemming from chronic systemic low-grade inflammation resulting in the aggravation of cardiovascular complications. An electronic literature search of PubMed and Embase up to March 2021 was performed for papers reporting the effects of tryptophan (TRP), kynurenine (KYN), kynurenic acid (KYNA), xanthurenic acid (XA), anthranilic acid (AA), and quinolinic acid (QA), focusing on their roles in carbohydrate metabolism and the cardiovascular system. In this review, we discussed the progress in tryptophan metabolism via KP research, focusing particular attention on the roles in carbohydrate metabolism and its complications in the cardiovascular system. We examined the association between KP and diabetes mellitus type 2 (T2D), diabetes mellitus type 1 (T1D), and cardiovascular diseases (CVD). We concluded that tryptophan metabolism via KP serves as a potential diagnostic tool in assessing cardiometabolic risk for patients with T2D.

## 1. Introduction

Tryptophan (TRP) is an essential exogenous amino acid that intermediates in human protein synthesis and has critical metabolic functions as a substrate for crucial molecules such as serotonin—(the neurotransmitter), nicotinamide adenine dinucleotide (NAD), and nicotinic acid [1]. TRP has been the object of numerous research because of its metabolism in a series of bioactive metabolites with the ability to influence many metabolic pathways of numerous cells in mammalian species. The kynurenine pathway (KP) is the hub of metabolism of peripheral TRP (95%) in humans and animals [2]. Additionally, it has been found that the KP plays an important part in several factors, such as immune activation, inflammation, and oxidative stress, which are associated with a variety of metabolic diseases [3].

Diabetes (Diabetes Mellitus—DM) is a heterogeneous group of chronic metabolic disorders that in combination result in hyperglycemia [4]. In data provided by the International Diabetes Federation, in 2019 approximately 463 million adults between 20–79 years (9.3%) had diabetes. It is forecast that in 2045, this number will rise to 700 million (10.9%). In 2019, 374 million people (7.5%) were estimated to be living with impaired glucose tolerance (IGT), which is a direct risk factor for developing diabetes. Furthermore, only in 2019, diabetes caused 4.2 million deaths and is one of the top ten leading causes of death [5], and DM represents by itself a major risk factor for cardiovascular events, which are the key cause of mortality in DM.

It has been increasingly evident that KP may be involved in the etiology of many chronic metabolic diseases, such as diabetes, metabolic syndrome, and atherosclerosis, which are well-known risk factors of cardiovascular diseases and increased mortality. A recently published review summarizes the role of KP in metabolic disorders including aging, atherosclerosis, obesity and diabetes [6]. The present literature review aims to provide an overview of the current findings of the involvement of kynurenine pathway metabolites in the pathogenesis of carbohydrate metabolism disorders and its associations with cardiovascular complications of diabetes.

Special emphasis was placed on presenting findings from either experimental and clinical studies on the role of particular KP downstream derivatives in carbohydrate metabolism. Moreover, a concise summary of the recent knowledge about KP alteration observed in atherosclerosis, heart failure and chronic artery disease is provided.

### Tryptophan Metabolism via the Kynurenine Pathway

**Figure 1:**
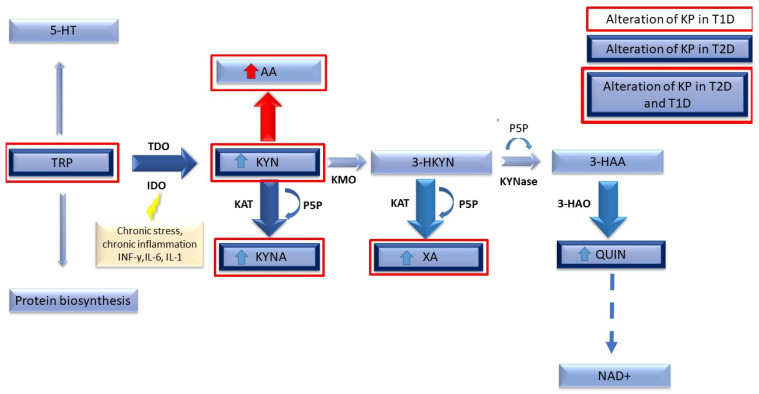
Schematic illustration of the kynurenine pathway (KP), including its serum alteration in type 1 diabetes (T1D) and type 2 diabetes (T2D). Dysregulation of KP in T1D was marked in red. Alterations of TRP catabolism along KP in T2D were marked in dark blue. Alterations of TRP catabolism along KP in T2D and T1D were marked in both red and dark blue. KYNase: kynureninase; 3-HAO: 3-Hydroxyanthranilic Acid Dioxygenase; P5P: Pyridoxal-5-Phosphate.

KP is the major TRP metabolic route in mammals. Many active metabolites, which are essential for the functioning of the organism, are produced through this pathway, resulting in NAD’s biosynthesis. Under physiological conditions, KP is found in almost all mammalian tissues, but mainly in the liver. The process begins when TRP, an exogenous amino acid, is converted to unstable N-formylo-L-kynurenine. This step is catalyzed by two independent enzymes belonging to the oxidoreductase family: tryptophan 2,3-dioxygenase (TDO) and indoleamine 2,3-dioxygenase (IDO) [7]. Then, N-formylo-L-kynurenine is turned into L-kynurenine (KYN) by the action of formaminidase enzyme. At this point, KYN can be converted into three different compounds by three enzymes: (1) kynurenine aminotranferase (KAT), which transaminates to kynurenic acid (KYNA); (2) hydrolyzed to anthranilic acid (AA) by kynureninase A; (3) hydroxylated to 3-hydroxykynurenine (3-HKYN) by kynurenine hydroxylase (KMO) [2]. Usually, the majority of kynurenine is converted to 3-HKYN and then converted to xanthurenic acid (XA) by kynurenine aminotransferase and to 3-hydroxyanthranilic acid (3-HAA) by kynureninase. This last compound is further converted to quinolinic acid (QA) by nonenzymic cyclization and contributes to the production of nicotinamide mononucleotide (NMN) and nicotinamide adenine dinucleotide (NAD), which plays a major role in redox reactions as a crucial cofactor and is significant in energy homeostasis. Figure 1 presents steps of the kynurenine pathway with marked alterations observed in serum during T2D and T1D (described in further sections).

## 2. Methods

This review summarizes clinical and experimental studies on the role of KP metabolites in diabetes and cardiometabolic risk factors (such as cardiovascular diseases, heart failure, and atherosclerosis). We conducted a comprehensive search of PubMed and Embase electronic databases retrieving the articles published between 1975 and 2020. The search included the terms “kynurenine pathway”, “tryptophan metabolism”, “indoleamine 2,3-dioxygenase”, “kynurenic acid”, “xanthurenic acid”, “3-hydroxykynurenine”, “3-hydroxyanthranilic acid” and “quinolinic acid” in combination with at least one of the following: “diabetes type 2”, “diabetes type 1”, “metabolic syndrome”, “obesity”, “coronary artery disease”, “heart failure”, “endothelium dysfunction”, “atherosclerosis”, “clinical study”, “in vitro”, “in vivo” and “experimental study”. We also searched references of the retrieved articles to locate other potentially eligible studies. After the initial analysis of the results, duplicate records, papers on neurodegenerative diseases, kidney diseases, bone metabolism, and cancers were removed. The studies analyzing the molecular background of the kynurenine pathway’s role in carbohydrate metabolism, its association with cardiovascular dysfunction, and the possible role of KP metabolites as biomarkers have been summarized.

## 3. Relevance of Tryptophan and Its Metabolites in Carbohydrate Metabolism

In recent years, a growing body of research-based data has indicated that tryptophan and KP metabolites are involved in alterations of carbohydrate metabolism.

In a study carried out as a part of the Shanghai Diabetes Study (SHDS), it was observed that serum TRP levels were significantly higher in patients who developed type 2 diabetes (T2D) in a 10-year follow-up compared to subjects who remained metabolically healthy. It was also observed that individuals with higher TRP levels presented more advanced insulin resistance (IR) and higher insulin secretion, elevated blood pressure, and triglyceride concentrations. The authors concluded that TRP can be regarded as a new marker associated with the risk of diabetes development in the Chinese population [8].

In turn, Yu E. et al. studied the associations of baseline and 1-year changes of these metabolites of TRP via KP with type 2 diabetes (T2D) incidence. They demonstrated that levels of baseline TRP, and after a follow-up period, increases in quinolinic acid (QA), were positively associated with the incidence of T2D. It was also shown during this observation that changes in TRP metabolites predicted alterations in HOMA-IR [9]. Furthermore, it was confirmed that TRP metabolism may be altered in patients with DM; and by measuring its metabolites, they showed lower levels of TRP in patients with DM than in nondiabetic subjects, which suggests that TRP metabolism may be accelerated. They showed that TRP appears to be more degraded by the KP when plasma TRP levels are high in DM. However, in patients with low TRP levels, the transformations through the serotonin pathway were intensified [10].

Changes in KP were also confirmed in a study of pregnant women in whom a significant increase in the urine concentration of most TRP metabolites was associated with gestational diabetes (GDM) in comparison to 34 healthy controls at every stage of pregnancy [11]. In this study, no correlation was found in maternal plasma levels of TRP and its metabolites between the groups of subjects. Interestingly, it was shown that the KP was activated before placental hormones or the fetoplacental unit could have produced any physiological effect.

In the other data conducted among 2519 individuals with coronary artery disease (CAD) without diabetes at baseline who were observed for a median of 7.6 years, plasma and urine kynurenine: tryptophan ratio (KTR) were evaluated. During the follow-up in 173 subjects (6.9%) a new incidence of T2D was identified. The urine KTR showed a strong positive association with incident type 2 diabetes. However, plasma levels of TRP and KP metabolites seemed to be similar to those in controls without T2D [12]. 

Experimental models help to understand the changes observed in humans. In animal studies, TRP was shown to regulate insulin and incretin hormones. Furthermore, it was observed that the addition of TRP to glucose load reduces glucose levels, probably by utilizing glucose via a glucose-mediated insulinotropic polypeptide [13]. A confirmation of these observations is the study by Lin et al., which showed that TRP stimulates the release of insulin from the islets of wild-type control (WT) mice in a dose-dependent manner in the presence of 11.1 mM glucose [14]. Besides, TRP activated the GPR142 receptor belonging to Gαq-coupled receptors with a role that has not yet been fully discovered. However, in the mouse model, GPR142 was confirmed to be a key mediator of TRP activity in insulin secretion [14]. It was proven that oral dosing of TRP inhibited peak glucose in WT mice during the intraperitoneal glucose tolerance test (IPGTT) and oral glucose tolerance test (OGTT) as well as rapidly and dose-dependent increased insulin, GIP, and GLP-1 plasma levels, all reaching maximum concentration 5–10 min after TRP administration [14].

Different observations were made in studies performed on non-obese and non-insulin-dependent Goto-Kakizaki (GK) diabetic rats fed a high TRP diet. On day 14 of the experiment, incremental blood glucose levels observed over 2 h (AAUC0–2 h) in rats fed a 3% and 5% TRP diet decreased by 13% and 18%, respectively, compared to GK control rats. However, no significant differences were found in the rats fed the TRP diet compared to the GK control rats. On day 28 of the experiment, there were no significant differences in the AUC0–2h blood glucose levels in any group, including the GK control group [15]. Furthermore, oral TRP supplementation to hereditary T2D rats helped to maintain proper glucose and insulin levels after oral glucose administration. What is interesting is that long-term feeding with tryptophan-enriched chow delayed the onset and progression of diabetes in this rat model. The probable mechanism that explains this phenomenon is the protective effect (of added TRP) from the exhaustion of pancreatic β-cells. Furthermore, it was reported that TRP itself can reduce the absorption of glucose from the small intestine [16] and a lack of this amino acid in the diet caused (reversible) a decreased tolerance to glucose [17]. The observation that TRP inhibits hepatic glucose production in the rat model, whereas in the liver of guinea pigs, TRP does not have any effect on gluconeogenesis, is noteworthy [18]. On the other hand, there are some data that showed that oral administration of TRP and its continuous use may not improve blood glucose in type 2 diabetic rats [19] and a diet high in TRP induces IR in pigs [20]. The discrepancy might be due to different mechanisms involved in carbohydrate metabolism in different species.

Based on the above reports, it is not possible to unequivocally assume the occurrence of an increase or decrease in TRP concentration in the course of carbohydrate disorders. Conducting studies in vivo models (including humans) is required because in vitro studies indicate that an increase in TRP concentration enhances insulin secretion, which may be the mechanism leading to IR.

Another issue that may bring new light to understanding the link between TRP transformation and glucose disorders is the relationship between kynurenines and arylate hydrocarbon receptors (AhR). The AhR is a ligand-activated nuclear receptor that regulates the expression of many genes. It is recognized as an important molecule in regulating immune response, including T-cell differentiation towards Th17 [21], stem cell maintenance, and cellular differentiation [22,23,24].

It was known to be activated by exogenous ligands, especially toxins (for ex. 2,3,7,8-tetrachlorodibenzo-p-dioxin-TCDD), until the KYN was discovered to be the first endogenous ligand [24]. As an agonist of AhR, KYN contributes to modulating the levels of reactive oxygen species (ROS) [25]. Moreover, AhR activation by KYN can induce IDO 1, which further promotes a positive feedback loop [26,27]. Recent studies indicate that KYN acts more like a pro-ligand requiring further “activation” to stimulate AHR. The authors based their theory on the observation that for this receptor to be activated, the KYN concentration must be at μM levels, compared with the pM/nM range of other ligands such as 2,3,7,8-tetrachlorodibenzo-p-dioxin (TCDD) and tryptophan photo-metabolite 6-formylindolo [3,2-b] carbazole (FICZ) [28,29]. However, in human plasma, KYN reaches concentrations in the order of µM.

The data indicate the involvement of AhR-kynurenine signaling in glucose and lipid metabolism. Biljes et al. observed higher glucose blood levels in AhR deficient mice [30]. On the other hand, Dabir et al. discovered for the first time that high glucose stimulation can rapidly activate AhR in aortic endothelial cells, which activates a potent anti-angiogenic and pro-atherogenic protein, trombospondin-1, and contributes to diabetic vascular complications [31].

What is interesting is that some human studies showed an association between exposure to AhR agonists (especially TCDD) and an increased risk of developing T2D and other metabolic disorders like hyperlipidemia or obesity [32,33]. Table 1 summarizes the most significant findings (in vitro and in vivo) and the effects that support the roles of kynurenines in carbohydrate metabolism.

### 3.1. Kynurenine (KYN)

KYN, the endogenous ligand of the aryl hydrocarbon receptor (AhR) transcription factor, is the first stable TRP metabolite with immunomodulatory properties [28]. It was shown that a receptor mechanism can promote differentiation of CD4 + effector T cells [34]. Like TRP, KYN easily crosses the blood–brain barrier (BBB) and its concentration in peripheral tissues affects brain concentration [7,35]. It was shown that over 60% of the central KYN amount is derived to the brain from the peripheral circulation [7,29].

KYN undergoes glomerular filtration and almost 100% reabsorption in the renal tubules at low plasma concentrations. However, when its concentration increases, as it was demonstrated in patients with T2D [36], KYN is eliminated to a greater extent by the renal route. This proves the strict regulation of clearance, depending on concentration KYN in plasma. The concentration of TRP metabolites depends on the supply of this amino acid in the diet, therefore, to observe its changes, the ratio of kynurenine:tryptophan (KTR) is used, which provides a more adequate assessment of TRP catabolism than the absolute concentration of KYN [12].

In patients with type 2 diabetes, the plasma levels of TRP and KYN were positively correlated with BMI, which may indicate a relationship between these compounds and obesity [37]. Acute exercise has been shown to directly affect circulating levels of TRP, KYN, and KYNA, suggesting that the KP supports the potential mechanism of exercise benefits to increase resistance to stress-induced depression [12].

It was observed that in patients with T2D one year after bariatric surgery, the levels of TRP and KYN metabolites decreased significantly and correlated with a decrease in usCRP [38]. However, there are slightly different reports, although confirming that obesity was associated with elevated plasma KTR levels, indicating a lack of normalization even after significant weight loss [12].

Evidence that stimulation of the immune system increases the production of KYN in diabetes is the result of experimental studies conducted on hepatocytes isolated from diabetic rats induced by streptozotocin. It was proven that incubation of these cells with [5-3H] L-TRP enhances the synthesis of KYN and QA [39]. On the other hand, another study showed that during the perifusion of freshly isolated, normal rat islets with a submaximal stimulating glucose concentration (11 mM), the addition of 0.1 mM KYN significantly increased insulin secretion. This demonstrates that not only in hepatocytes, but also in pancreatic islets, glucolipotoxicity and inflammation may contribute to the activation of the KP. The modulation of KP in these organs might therefore be considered potential targets for novel therapies [40].

Taking the above into account, the concentration of KYN as a compound with immunomodulatory properties correlates with the degree of stimulation of the immune system and in many cases, though not always, with the degree of obesity. Undoubtedly, the high concentration of KYN observed in the course of diabetes contributes to the release of insulin.

### 3.2. Kynurenic Acid (KYNA)

Despite the fact that the literature about the exact impact of KP on carbohydrate regulation under physiological conditions is sparse, the available data indicate that KYNA can increase glycemia by autonomic regulation, while inhibiting proinsulin synthesis [41]. This compound is classified as an N-methyl-D-aspartate (NMDA) receptor antagonist. The literature data indicate that compounds with this mechanism of action have antidiabetic properties that increase the function and survival of beta cells [42]. Additionally, literature data show that antagonists of NMDA receptors inhibit glucose production induced by NMDA agonists injected into the vagal dorsal nerve complex in rodents. Moreover, it has pro-oxidizing properties [43]. Furthermore, KYNA is a compound that activates the Gpr35 (G protein-coupled receptor). According to recent reports, this mechanism enhances metabolism in adipose tissue and leads to diminishing weight gain in animals fed a high-fat diet and improving glucose tolerance [44].

The serum concentrations of both KYN and KYNA were higher in proliferative diabetic retinopathy (PDR) patients when compared to non-proliferative (NPDR) patients, pointing to the potential contribution of both of these compounds to the pathogenesis of diabetic retinopathy [45]. Interestingly, changes in KP metabolite concentrations can also be observed not only in urine and serum, but also in the saliva of diabetic patients. One study showed significantly elevated KYN and KYNA concentrations in patients with the earlier diagnosed complication of diabetes in the form of gingivitis and periodontitis [46]. Accelerated TRP metabolism can be linked with changes in the oral microorganism profile and chronic inflammation in the periodontal pocket observed in the course of especially hypertensive T2D patients [47]. Experimental studies showed that in the course of diabetes there is an increase in the urinary excretion of KYNA [48]. Moreover, it was observed that KYNA, like XA, preferentially inhibits proinsulin synthesis in isolated rat pancreatic islets [41]. KYNA belongs to the NMDA receptor antagonists. Literature data show that antagonists of these receptors inhibit glucose production induced by NMDA agonists injected into the vagal dorsal nerve complex in rodents [49]. Thus, there are clear indications that KYNA, by centrally regulating glycemia as well as by inhibiting insulin synthesis, plays an important role in carbohydrate metabolism, undoubtedly creating conditions conducive to the impairment of these processes.

### 3.3. Xanthurenic Acid (XA), 3-hydroxykynurenine (3-HKYN), 3-hydroxyanthranilic Acid (3-HAA), and Quinolinic Acid (QA)

Increased urinary XA excretion was demonstrated in patients with T2D diabetes compared to healthy individuals [50]. Besides the elevated expression of IDO and an increase in the levels of XA, KYN, and 3-HKYN in the serum of patients with diabetic retinopathy was observed [45]. This observation indicates that TRP metabolites play a key role and can most likely be involved in the pathogenesis of diabetic retinopathy by exacerbating oxidative stress. Of all metabolites of the KP, only QA and XA were significantly associated with improved glucose control in diabetes patients after bariatric surgery. It was proved that the one-year QA concentration was negatively associated with the fasting glucose and HbA1c, whereas the XA delta correlated positively with the insulin sensitivity index [38].

Moreover, other KP metabolites, such as 3-hydroxykynurenine (3-HKYN) and 3-hydroxyanthranilic acid (3-HAA), inhibit leucine-induced insulin release from pancreatic islets isolated from rat pancreas [51]. It is also worth mentioning that one of the first data examining the connection between KP and T2D proved in vivo in a rat model that xanthurenic acid (XA) can impair the action of insulin by binding to its circulating fraction and then the whole complex combines to the anti-insulin serum [52]. In a pre-clinical study, XA was used as a diabetes inducer in rats [53]. There are four potential mechanisms responsible for XA pathogenesis. (1) The formation of insulin chelate (XA-In) complexes having antigenic properties and 49% less activity than pure insulin [53]; (2) The formation of insulin-Zn ++ complexes in β cells that are toxic to isolated pancreatic islets [54,55]; (3) The inhibition of insulin secretion from rat pancreas [16]; and (4) The initiation of pancreatic beta-cell apoptosis through a caspase-3-dependent mechanism and destruction of mitochondria and nuclei [55,56].

QA is an NMDA receptor agonist responsible for excitatory action in CNS [7,43]. However, its presence was also recently proven in pancreatic cells. These receptors reduce the amount of insulin secreted by β cells in response to stimulating glucose levels. NMDA receptor deletion in mouse islets was proven to increase glucose-induced plasma insulin levels and lower blood glucose levels. In inflammation, as well as in the course of carbohydrate disorders, the death of β-cells with NMDAR expression was observed [42]. In the PREDIMED trial, it was observed that the annual increase in QA, like the baseline TRP, was positively associated with T2D incidence. Besides, both baseline and annual changes in TRP metabolites predicted changes in HOMA-IR [12]. It was demonstrated in a further study that after exposure to QA, the serum level of glucose and total cholesterol was significantly reduced [57].

## 4. Tryptophan Metabolism in Type 1 Diabetes Compared to Type 2 Diabetes

Far too little is known about disturbances among down-stream kynurenine/tryptophan metabolic shunt in patients with T1D (Type 1 Diabetes—T1D). Although there are some limited but significant data, there are some significant differences between the concentrations of the individual pathway metabolites between patients with T1D and T2D. T1D is associated with significantly elevated levels of anthranilic acid (AA), which has not been observed among patients with T2D in any study yet. Moreover, levels of KYNA and XA are more elevated than previously reported in patients with T2D and healthy ones. Additionally, decreased KTR has been observed in T1D. It is suggested that this specific shift of KP, which leads to excessive production of AA, may be the effect of a vitamin B2 deficiency. Riboflavin is considered to be a cofactor of the enzymatic transformation of KYN to 3-HKYN. Therefore, as a result of its deficit, this metabolic route is changed into a KYN-AA formation. What is interesting is that, in patients with T2D, these alterations of riboflavin serum level have not been observed [63]. These results were in line with the previous study that showed a lack of vitamin B2 in the majority of patients with T1D, but no deficiency was found in healthy ones [64] [Figure 1]. Furthermore, metabolome research conducted on children with T1D revealed elevated TRP catabolites (KYN) in the urine [65].

The impaired immune response and inflammation are the key mechanisms mediating the relationship of TRP transformation via KP and diabetes. IDO (the enzyme catalyzing the first rate-limiting step in KP) is supposed to be the binding factor, which plays its role in dendritic cells.

Many studies showed that IDO is responsible for immunoregulatory tryptophan catabolism. This enzyme is capable of modulating immune cell activation status and phenotype via several molecular mechanisms [66]. The expression of IDO, especially in dendritic cells (DC), has a strong immunomodulating effect and it can induce either immune activation or tolerance depending on the current environmental conditions [67]. DCs are the main antigen-presenting cells, playing an important role in the initiation of autoimmune diabetes in NOD mice (a prototypic animal’s model for autoimmune T1D) islet cells [68,69]. In the experimental study, Grohmann U. et al. demonstrated that impaired expression and activity of IDO in pDCs (plasmacytoid dendritic cells) reveals a poor response to IFN-γ stimulation (the principal IDO inducer in vitro and in vivo) and impairs the development of immune tolerance to autoantigens in NOD mice [70]. Additionally, it was observed that increased IDO expression in both pancreatic lymph nodes and bone marrow-derived dendritic cells has a protective effect against T1D development [71]. Additionally, Orabona et al. demonstrated that T1D in children was characterized by a remarkable defect in IDO1 function in sera and peripheral blood mononuclear cells in comparison with the control group [72].

Another recent study described, for the first time in humans, a defect (significantly decreased or even absent) of IDO1 expression in pancreatic β-cells from patients with T1D. The decreased IDO expression was also observed in donors without a diabetes diagnosis, but with multiple positive autoantibodies (AAb+). This finding could indicate that loss of IDO function is present before illness begins (in pre-diabetes of T1D) and could lead to developing diabetes. These new insights imply the possible IDO-1 involvement in the early stages of islet dysfunction [73]. It was observed that TRP derivatives may play an important role in neuronal damage development in T1D. Chmiel-Perzyńska et al. demonstrated significantly increased hippocampal KYNA concentrations during DM, either untreated or treated with insulin [74]. Furthermore, experimental data shows that IDO-2 and kynurenine 3-monooxygenase (KMO) are important in neuropathic pain pathology and their inhibition can effectively reduce neuropathic pain [75]. Elevated IDO and QA can contribute to the development of diabetic retinopathy characterized by neuronal degeneration, because their increased expression was observed in both human and rodent retinas [76]. QA is a well-known excitotoxin, whose chronic excessive production via altered KP results in neuroinflammation [77].

These observations put a new light on the pathogenesis of T1D and in the future could contribute to discovering new drugs able to target IDO expression in pancreatic β-cells. However, the available data suggest that the KP could be involved not only in the pathogenesis of diabetes, but also in the development of its complications.

## 5. Tryptophan Metabolism in Type 2 Diabetes

Patients with T2D have higher levels of urine TRP and KYNA. Similar observations were established in patients with earlier developed diabetic retinopathy [45]. Extensive research conducted by Meyramov G. et al. revealed that some KP metabolites can form chelate complexes with insulin, which directly reduces insulin activity by up to 50%. What is interesting is that, in regular laboratory tests, normal and chelate insulin are indistinguishable [78]. Moreover, it was proven that under physiological conditions, some of the TRP/KYN pathway (TKP) genes are constitutively expressed in rat islet cells (TDO2, KMO, Kase, KAT1-KAT4) [40]. The only exception is transcripts of IDO and ACMSD (enzyme aminocarboxymuconate-semialdehyde-decarboxylase regulating and limiting the formation of QA). Changes in the transcript of these genes were observed after exposition to IFN-γ. These findings are in line with previous research that pancreatic islet cells are also affected by inflammation during obesity and T2D [79].

Several studies on humans confirm that when pancreatic islets are exposed to IFN-γ, increased levels of transcripts encoding IDO were observed following an increase in KYN concentration [79,80]. This supports the findings and points to a strong contribution of inflammatory factors in the pathogenesis of type 2 diabetes [81]. Under this condition, insulin secretion assessed by glucose-induced insulin secretion (GSIS) is impaired, but the data of Liu et al. reported that it can also be improved by the supply of KYN (to normal islets) [40]. It could be a probable protective mechanism that is intended to protect the pancreatic islets from damage caused by the cytotoxic activity of the immune system.

A study carried out on streptozotocin-induced diabetic rats confirmed the involvement of the KP in the control of glucose-induced insulin secretion and hepatic glucose production in the state of T2D. TRP, KYN, and KYNA administration significantly increased insulin secretion from the diabetic isolated pancreas in response to glucose. Furthermore, the same KP metabolites (TRP, KYN, KYNA) in the case of the absence of glucose, induced a glucagon release (the second hormone produced by islet cells, antagonist to insulin). It was shown that in the hepatic cells of non-diabetic Wistar rats, the supply of TRP and KYN inhibited hepatic glucose production [82]. This data suggests that TRP transformations leading to KYNA formation via indirect KYN may be a protective mechanism in diabetes.

On the other hand, it was also established that the transformation of TRP through the KYN-3-HKYN-XA pathway promotes the formation of metabolites that were more diabetogenic, described in the literature as the “kynurenine hypothesis of insulin resistance and its progression to T2D” [83]. Recently published study results fully support the “hypothesis” that indicates the overproduction of diabetogenic KP metabolites induced by chronic stress and low-grade chronic inflammation, which is crucial to the mechanisms promoting the development of T2D [84]. It is postulated that KP metabolites can serve as T2D biomarkers and future targets for clinical intervention.

Those studies provide a well-supported but intricate interplay between TRP and its metabolites and the development of T2D. Interestingly, some research has discovered decreased TRP levels and higher KYN/TRP ratios in pre-diabetes [85,86]. Further work should be undertaken to explain those differences.

**Figure 2:**
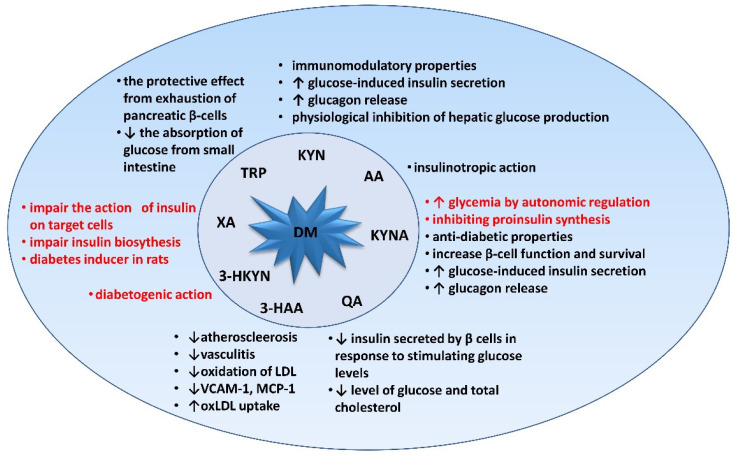
The biological behaviors of kynurenine pathway metabolites and their contribution in metabolic syndrome and T2D. KP appears to be one of the important factors regulating the mechanisms involved in the development of T2DM in the pre-diabetic state.

The involvement of KP metabolites in the pathogenesis of diabetes mellitus is presented in Figure 2.

## 6. Linking Cardiovascular Diseases to Diabetes and the Kynurenine Pathway

Many years of research have shown that the probability of death from cardiovascular diseases (CVD) in patients with carbohydrate metabolism disorders is comparable to that of people with coronary artery disease (CAD) after myocardial infarction, but without diabetes [87]. The risk of vascular outcomes, including ischemic stroke, vascular deaths, and coronary heart disease, is twice as high in patients with diabetes [88]. CVD is responsible for approximately half of the deaths among patients with T2D, which makes it the leading cause of mortality in this group [89]. However, the most common primary symptoms of cardiovascular complications in T2D are heart failure and peripheral vascular disease [90]. 

It has been well established that low-grade chronic inflammation, immune activation, and oxidative stress (SOX) are pivotal factors in the pathogenesis of atherosclerosis and CVD. Additionally, there is increasing evidence that the KP has drawn considerable attention to the above-mentioned contributing factor. Accelerated TRP degradation via the kynurenine pathway occurs during immune system activation [91,92]. Notably, the contribution of KP’s role in chronic inflammation as a factor of atherosclerosis development and progression has been well documented [34,93].

The link between the immune system and the kynurenine pathway is INF-γ as well as IL-6 and IL-1 [94,95]. These cytokines have been assigned a crucial role in the induction of IDO activity. The increase in TRP degradation via the KP has been proven in patients with confirmed ischemic heart disease [96,97] and atherosclerotic lesions in peripheral arteries [98]. One of the KP metabolites, 3-hydroxyanthranilic (3-HAA), has been presented as an immune regulatory compound that plays its role by inhibiting T cell responses and increasing the number of Tregs [99,100]. Furthermore, some studies imply its significant role in reducing atherosclerotic lesion size in arteries combined with reduced T-cell driven vascular inflammation and decreasing plasma lipid levels (especially VLDL, total cholesterol levels, and TG) [101]. Further investigation conducted by the same study group confirmed IDO involvement in atherosclerotic plaque formation and vascular inflammation. The inhibition of IDO contributed to the remarkable aggravation of atherosclerosis in the aorta by up-regulating VCAM-1, CCL2, and rising CD68 macrophage accumulation in the lesions [102]. These findings were in agreement with the observations of Cole et al., who also demonstrated that loss of IDO resulted in decreasing IL-10 production and modulating plaque composition towards increased macrophage content [98].

A strong positive correlation between IDO activity and neopterin concentration [103], a marker of inflammatory process activity considered a risk stratification factor for cardiovascular events in patients with coronary artery disease and the occurrence of left ventricular dysfunction in patients with angina pectoris, was also observed [104,105]. Moreover, it was proven that vascular endothelial cells can synthesize de novo KYNA from a bioprecursor such as KYN [106,107]. Another study revealed that KYN can also be considered a vasoactive compound that plays its role via the sGC-cGMP-dependent protein kinase pathway [108] in the endothelium and via the activation of the Kv7 channels (voltage-dependent K(+) channels encoded by the KCNQ gene family) in vascular smooth muscles [109]. Further investigation recognized another KP metabolite, XA, as a more efficacious vasodilator than KYN, but the exact molecular mechanism remains unclear and requires elucidation [110].

This information leads one to consider whether plasma kynurenines can be a new risk factor for cardiovascular diseases. Moreover, the data suggest a correlation between early enhanced activation of the KP and poor outcome after post-cardiac arrest shock [111]. Eussen et al. demonstrated that KYN and 3-HKYN are associated with an increased risk of acute coronary events and suggest that the KYN route is involved in the development of ischemic heart disease at the initial stages. Furthermore, the results of this large prospective study show that high plasma KYN and 3-HKYN concentrations are associated with an increased risk of acute coronary incidents in the elderly population [112]. In patients with suspected stable angina pectoris, elevated plasma KYN concentrations increase the risk of acute myocardial infarction [113].

## 7. Atherosclerosis, Endothelium Dysfunction, and the Pathogenesis of Coronary Artery Disease

In studies conducted on a mouse model, one of the KP metabolites, 3-HKYN, was shown to accelerate endothelium apoptosis and as a result its dysfunction by/via NAD(P)H oxidase up-regulation. This happens because of enhanced superoxide anion production mediated by this enzyme, which promotes oxidation stress in the cells [114]. On the other hand, XA which is a KAT-derived metabolite of 3-HKYN was found to possess potent antioxidant capabilities [115].

Consequently, the 3-HKYN/XA ratio could be considered an issue reflecting the disturbed balance between cell apoptosis and antioxidation properties in the endothelium.

Another aspect that requires to be highlighted is the role of 3-HAA in atherogenesis. It is suggested that 3-HAA plays a role in the control of autoimmunity through a direct influence on T cells and increased antigen presentation by macrophages and dendritic cells. In the literature, 3-HAA has been presented as an immune regulatory compound, which plays its role by inhibiting proinflammatory Th1 and Th2 cells while increasing the number of Tregs. Research on the autoimmune rodent encephalitis model revealed that 3-HAA reduced the inflammation triggered by Th17 cells [116]. Another study conducted on a mouse model of asthma reported that 3-HAA can inhibit NF-kappaB, resulting in the death of previously activated Th2 cells [99]. 3-HAA has been demonstrated to be a compound with properties reducing the expression of the vascular cell adhesion molecule (VCAM)-1, which mediates the adhesion of immunological cells to the vascular endothelium and plays an important role in atherosclerosis development [117]. Moreover, this compound is known for decreasing the secretion of monocyte chemoattractant protein (MCP)-1 in the endothelium, the chemokine responsible for the recruitment of monocytes, T cells, and dendritic cells to the site of inflammation [118].

Zhang et al. reported a significantly decreased oxLDL uptake by macrophages in 3-HAA-treated mice, showing the direct influence of this compound on macrophages. Furthermore, they demonstrated that treatment with 3-HAA led to a significant reduction of CD4+ T cell infiltration in the plaques, increased HDL concentration by stimulating the expression of ApoAI and ABCA1 genes located in the liver, lowered plasma cholesterol and triglyceride levels, and finally reduced local inflammation in vessels [101]. Interestingly, the authors suggest that the possible mechanism of 3-HAA’s action on the immune response of an organism is based on its action on peripheral immune cells rather than its effect on bone marrow. Cole et al. also confirmed that orally administered 3-HAA (3,4-DAA 3,4-dimethoxycinnamoyl anthranilic acid) contributed to the reduction of vascular inflammation and the size of atherosclerotic plaques [98]. Additionally, the role of quinolinic acid (QA), a metabolite of 3-HAA, has been emphasized in many studies. It was found that this metabolite plays a very important role in the dysregulation in the oxidant/antioxidant ratio, and increases the SOX in two ways: (1) by causing mitochondrial dysfunction activating NMDA receptors [119] and (2) increasing free radicles formation [120].

### 7.1. Role of IDO in the Progression of CVD

Two oxygenases (IDO and TDO) involved in the first and the most important reaction of KP differ in their site of action as well as the factors that activate them. Initially, it was thought that TDO occurs only in the liver in the cytosolic fraction, but recent studies indicate its presence in the epididymis, the testis, the placenta, the pregnant uterus, and the brain [121,122]. The presence of the second enzyme, IDO, was demonstrated in the cytosolic fraction of extrahepatic tissues like the kidneys, spleen, intestine, brain (especially in the hypothalamus), placenta, epididymis, endocrine glands, and monocytes. Nowadays, it is well established that IDO is an important link between the KP and immunoinflammatory response [123,124]. KP metabolites are commonly referred to as anti-inflammatory particles, which also possess the ability to affect auto-tolerance mechanisms [125]. IDO expression takes place in endothelial cells of the vessel wall, the leukocytes present inside them, and smooth muscle cells. This process is stimulated by proinflammatory cytokines such as TNF, IFN-γ, or LPS. It was confirmed that INF-γ, which is a cytokine synthesized by T lymphocytes, influences the regulation of the atherosclerotic plaque [126]. It is being suggested that IDO fulfills an atheroprotective role. The research showed that IDO inhibition using 1-methyl-TRP (1-MT) when given orally resulted in a statistically relevant increase in VCAM-1 expression in tunica media and increased CD68+ macrophage infiltration into the intimal part of the arterial wall of Apoe-/- mouse. This process leads to a relevant increase in the inflammatory process and the consequence of atherosclerotic plaque development progression. Subsequently, it was verified that IDO repression has an upregulating effect on VCAM-1 protein in the SMC cells grown from human coronary vessels. It was proven that this effect can be reversed using 3-HAA, which was confirmed in previous studies conducted by those authors [102].

Another way in which IDO may influence the progression of atherosclerotic plaque is its ability to induce the immunomodulating functions of B lymphocytes. Its action led to reduced amounts of IL-10 being synthesized by B lymphocytes, which are wildly known for their protective effect in the progression of atherosclerosis [103]. Furthermore, it was proven that in the absence of IDO, there is increased infiltration of CD68+ macrophages and CD4+ T lymphocytes. On top of that, reduced amounts of SMC have been noted in the atherosclerotic plaque. What is interesting is that a lack of IDO resulted in increased expression of GATA3 (transacting T-cell specific transcription factor 3), which suggests the facilitation of the Th2 response. The IDO deficiency led to reduced KYN concentrations, while it did not affect TRP concentrations in both IDO positive and negative subjects [120]. 

Those observations are in line with the data gathered as part of the Tampere Vascular Study, in which enhanced expression of IDO was observed in atherosclerotic plaque highly infiltrated by macrophages [100]. KYNA inhibits the release of TNF and high mobility group box protein 1 (HMGB1) through monocytes and granulocytes [127].

### 7.2. The Kynurenine Pathway Links Inflammation, Immune Response to Heart Failure 

An important role in the pathogenesis of heart failure (HF) has been assigned to monocyte-derived macrophages [128]. Their activation stimulates myocardial fibrosis resulting in unfavorable remodeling of the myocardium [129,130], simultaneously inducing an increased transformation of TRP via the KP. This leads to an accumulation of TRP metabolites in the heart tissue, which is known for its immunological and oxidative properties. Additionally, in both experimental studies and clinical trials on individuals with HF, elevated pro-inflammatory cytokines [131], including INF-γ, which is well known for inducing IDO activity, were observed. According to the research conducted by Jones et al. on human peripheral mononuclear cells (monocytes) treated with INF-γ, significant up-regulation of IDO-1, as well as raised KTR and elevated blood concentration of KYN, 3-HKYN, and QA, were detected, which in contrast did not occur in the INF-stimulated lymphocytes [132]. KTR is considered to be a marker of the cellular immune response, which can reflect the activation of monocytes [133]. Lund et al. hypothesized that myocardial fibrosis, which plays a crucial role in HF progression, could be linked to the excess formation of KP metabolites by activated monocytes [134]. The same study demonstrated the relationship between the increased concentration of kynurenine pathway metabolites (3-HKYN, QA, and XA) and increased mortality in patients with heart failure independent of coexisting CAD [134]. Another recent study reported an association between elevated kynurenine plasma levels and increased NT-proBNP about NYHA class. The severity of CHF correlated with the rate of KYN concentration. Interestingly, KYN (not NT-proBNP) was shown to be a better prediction factor of death among patients with previously diagnosed CHF than the well-known CHF biomarker NT-proBNP [135].

**Figure 3:**
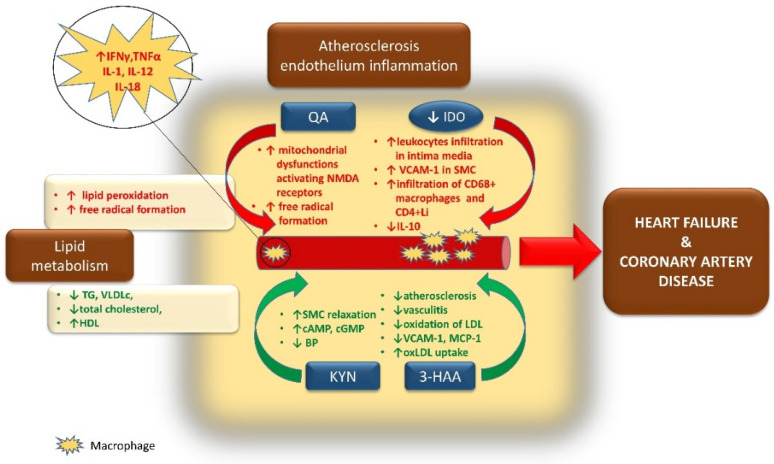
The involvement of kynurenine pathway metabolites in the pathogenesis of heart failure and coronary artery disease.

Although elevated KYN plasma levels in HF patients are well established, it was shown that KYN concentration differs significantly among patients with HF depending on the coexisting complications. In a longitudinal observation study, SICA-HF higher KYN values among patients with HFrEF were detected in those with coexisting arterial hypertension and atrial fibrillation as well as in a group with HFpEF in those with ischemic etiology. It was also observed that having higher KYN concentrations resulted in a higher risk of death [136]. Figure 3 summarizes the role of KP metabolites in cardiovascular complications.

## 8. Possible Avenues for Therapeutic Action and Conclusions

It is now clear that TRP metabolism via the kynurenine pathway has multiple important roles in the body, and the increasing research effort in this area is gradually revealing the intricate derivatives of the kynurenine-mediated interplay between diabetes, metabolic disorders, and cardiovascular complications. Although many issues remain hazy, in the presented review of the literature, we outline some potential prospects for therapeutic action. 

An impressive amount of data on the reduction of inflammation by some metabolites of KP is available. It might be conceivable to control IDO activity and the amount of bioavailable circulating 3-HAA, and hence to modulate low-grade diabetes-induced inflammation. 

Alternatively, it might be possible to enhance endogenous KYN, KYNA production to use their anti-diabetic properties. 

Additionally, in T1D, there is some promising suggestion that by restoring IDO function normoglycemia can be restored (for instance by using IL-6 receptor blockers), as was suggested by Orabona C. et al. [72]. It cannot be ruled out that kynurenine derivatives may indirectly become a premise for a completely new concept of diabetes therapy. There are numerous indications that an in-depth exploration of changes in TRP metabolism would accelerate our understanding of the pathogenesis and course of diabetes, contribute to the prevention, early detection, and more effective treatment. The rate at which the role of KP is being clarified in developing DM and its CVD complications makes it certain that in the future TRP metabolism can be pivotal to pharmacotherapeutic approaches to diabetes-induced inflammatory complications in the cardiovascular system.

Understanding the actions of TRP metabolism, as well as the mechanisms regulating the activity of KP enzymes, is extremely important, but still too incomplete to generate well-supported therapeutic hypotheses. If this could be achieved, it would undoubtedly have multiple benefits for personalized and precise diabetes medicine.

Derivatives of the kynurenine pathway (especially KYN, KYNA, XA), because of their ability to interact with specific receptors (such as AhR, NMDAR, Gpr35) expressed in tissues relevant for carbohydrate metabolism (pancreas, adipose tissue, liver), can be considered as modulators of metabolic disorders. Moreover, enhanced IDO expression is observed in atherosclerotic plaques in vascular inflammation, as well as in β-cells in diabetes. According to reviewed literature, IDO should be considered as a novel factor linking diabetes and cardiovascular diseases.

## Figures and Tables

**Table 1:** Summary of changes in TRP and its metabolites associated with impaired carbohydrate metabolism and its influence on carbohydrate metabolism in vitro and animal models.

Kynurenine Pathway Metabolite	Effect/Changes	Comments	References
**TRP**	Across the aging time course increase in the concentration of tryptophan.	The non-obese diabetic (NOD) inbred mouse strain recapitulates the autoimmune nature of T1DM, the NOD-E (transgenic NOD mice that express the I > E heterodimer of the major histocompatibility complex II).	[48]
	Tryptophan stimulates the release of insulin from β cells of the pancreas and incretin hormones via GPR142 signaling.	Wild-type control (WT) mice in a dose-dependent manner in the presence of 11.1 mM glucose.	[14]
	Tryptophan deficiency in the diet can modulate glucose tolerance.	Feeding rats for 14 days with TRP deficient diet caused worse glucose tolerance, and it was reversible after feeding a complete diet.	[17]
	Surplus tryptophan in the diet induces insulin resistance.	Feeding pigs for 3 weeks with a high (13.2%) vs. normal (3.4%) TRP large neutral amino acids (LNAA) diet.	[20]
	Enhanced TRP disappearance from the bloodstream in diabetic rats after a tryptophan load Impaired acute accumulation of TRP in the diabetic rat brains.	Normal vs. streptozotocin-diabetic rats.	[58]
**KYN**	“Acute exposure to KYN” enhances glucose-induced insulin secretion.	Normal rat islets and the INS-1 β-cell line.	[40]
**KYNA**	Increase in the concentrations of KYNA observed with the progress of T1D.	NOD vs. NOD-E mice.	[48]
	Fifty percent higher concentrations of KYNA in the serum from Zucker fatty rats.	Leptin-receptor-deficient Zucker fatty rats (ZFR)(fa/fa) vs. age-matched lean rats (FA/-).	[28]
	A 1,8 fold increase in the concentration of KYNA in urine od T2D nonhuman.	Normal vs. spontaneously and naturally diabetic nonhuman primate (rhesus macaques).	[59]
	KYNA inhibit the pro-insulin synthesis.	Isolated rat pancreatic islets, KYNA in millimolar concentrations.	[41]
**XA**	Increases urine excretion of XA as its complex with Zn2+.	Alloxan- and streptozotocin-induced diabetic rats.	[55]
	Forms complexes with insulin and reduces insulin activity.	XA-induced diabetic rats.	[53]
	Inhibits pro-insulin synthesis.	Isolated rat pancreatic islets; XA in millimolar concentrations.	[41]
	Induces pancreatic β-cells death.	Damage caused probably via caspase-3 dependent mechanism.	[49]
**3-HKYN** **3-HAA**	Inhibit the leucine stimulated release of insulin at concentrations below 10 mM.	Isolated pancreas islets from rats.	[51]
**QA**	Inhibitory effect of QA on phosphoenolpyruvate carboxykinase activity in diabetic rats; in vivo studies showed that intraperitoneal injection of 300 mg/kg^−1^ b.wt. initiates reduction of blood glucose level in 1 h after injection, restoring the blood glucose to its normal level at 2 h postinjection and keeping it constant for at least a further 4 h.	Streptozotocin-induced diabetes in rats; in vitro studies in rat’s liver.	[60]
	QA-induced increase of brain glucose uptake (55%), (14)CO_2_ generation from glucose, acetate, and citrate was inhibited (up to 60%). QA provokes a mild impairment of brain energy metabolism in vitro.	Thirty-day-old rats.	[61]
	STZ-diabetes mellitus causes augmentations of both KYN and QA generations in the liver, indicating the possibility that the immune and neuronal systems of insulin-dependent diabetes mellitus would be influenced by the increased amounts of KYN and QA.	Streptozotocin-induced diabetes in rats; the hepatocytes isolated from the rats were incubated with [5-3H]L-TRP.	[39]
	QA expression in the retinas of diabetic rats could contribute to the neuronal degeneration that is characteristic of diabetic retinopathy.	Streptozotocin-induced diabetes in rats.	[62]

## Data Availability

Not applicable.
